# Identification of Reliable Reference Genes under Different Stresses and in Different Tissues of *Toxicodendron succedaneum*

**DOI:** 10.3390/genes13122396

**Published:** 2022-12-17

**Authors:** Dongxiao Ma, Qin Zhang, Jintao Zhou, Yu Lu, Xiaomeng Duan, Chengzhong He, Jinde Yu

**Affiliations:** 1 College of Life Sciences, Southwest Forestry University, Kunming 650224, China; 2 Key Laboratory for Forest Genetic and Tree Improvement and Propagation in Universities of Yunnan Province, Southwest Forestry University, Kunming 650224, China; 3 Key Laboratory of Biodiversity Conservation in Southwest China, State Forestry Administration, Southwest Forestry University, Kunming 650224, China

**Keywords:** gene expression, reference gene, RT-qPCR, *Toxicodendron succedaneum*

## Abstract

*Toxicodendron succedaneum* (L.) Kuntze (*T. succedaneum*) is an economic tree species that produces urushiol and urushi wax, and it is of great value in industry and medicine. However, the stability of reference genes (RGs) has not been systematically reported in *T. succedaneum* to date. In this study, the expression of 10 candidate RGs was analyzed by RT-qPCR in different tissues (roots, stems, leaves), stress treatments (high/low temperature, drought), and hormone stimulation (jasmonic acid, JA). Then, the stability ranking of 10 candidate genes was evaluated by ∆Ct analysis and three software programs: geNorm, NormFinder, and BestKeeper. Finally, RefFinder was used to comprehensively analyze the expression stability of 10 candidate genes. The comprehensive analysis showed that *TsRG05/06*, *TsRG01/06*, and *TsRG03/ACT* were stable under high/low-temperature stress, drought stress, and JA treatment, respectively. *TsRG03* and *ACT* had stable expression in different tissues. While the *TsRG03* and *ACT* were recommended as the suitable RGs for *T. succedaneum* in all samples. Meanwhile, *UBQ* was the least suitable as a reference gene for *T. succedaneum*. In addition, the results of geNorm showed that the combination of two stable RGs could make the results of gene expression more accurate. These results provide alternative RGs for the study of gene function, correction, and normalization of target gene expression and directed molecular breeding in *T. succedaneum*.

## 1. Introduction

Reference genes (RGs), also known as housekeeping genes, are stably expressed in various cells, and are less affected by external factors [1,2]. RGs are widely used in molecular biology research, such as RT-qPCR, ribonuclease protection assay (RPA), northern blotting, gene chips, Western blotting, etc. [3,4,5]. The stability of RGs affects the evaluation of gene expression levels and the accuracy of quantitative analysis [6,7]. Therefore, the appropriate RGs should be selected for data standardization and error correction of target gene expression. Most of the commonly used RGs, such as *ACT* (actin), *TUB* (tubulin), *UBQ* (*ubiquitin*), *GAPDH* (glyceraldehyde-3-phosphate dehydrogenase), *18S rRNA* (18S ribosomal RNA), *28S rRNA* (28S ribosomal RNA), *PP2A* (protein phosphatase 2A), and *EF-1α* (elongation factor 1-alpha), are the housekeeping genes that constitute the cytoskeleton or maintain the essential metabolism required for the normal life of cells [7,8,9,10,11,12]. In addition, some studies have shown that many newly identified genes, including *RPII* (RNA polymerase subunit), *UNK1* (hypothetical protein), *F-box* (F-box protein), and many small RNAs, are more suitable for RGs than some traditional genes [13,14,15,16,17].

However, a handful of studies have confirmed that plants lack universal RGs, and the applicability of specific RGs depends on experimental conditions and plant species [18,19,20,21]. Accordingly, the use of RGs with unstable expression may result in biased results and false positives [22,23]. For example, several commonly used RGs (*TUB*, *ACT*, *UBQ*, and *EF-1α*) were found to be unstable in different tissues of *Arabidopsis thaliana* (*A. thaliana*) and hybrid aspen (*Populus tremula* × *Populus tremuloides*) [24]. *EF-1α* is the most stable reference gene in potatoes under late blight and salt stress, while *EF-1α* and *APRT* (adenine phosphoribosyltransferase) are the most stable RGs under cold stress [12]. Similarly, the expression of *β-tubulin* was not stable during fruit development in cherry (*Cerasus pseudocerasus*) [25]. Therefore, the selection and validation of RGs under diverse experimental conditions is crucial to obtaining reliable quantitative analysis results.

*T. succedaneum* belongs to the family Anacardiaceae, a deciduous and dioecious tree [26]. The lacquer wax produced from its seeds can be used in the production of cosmetics, waterproofing agents, coatings, adhesives, lubricants, etc. [27]. Meanwhile, *T. succedaneum* has important applications in the medical field; for instance, modern medicine has found that laccase and urushiol of *T. succedaneum* can be used as cancer inhibitors [27]. Furthermore, its well-developed root system can be used to reduce soil and water loss; therefore, *T. succedaneum* plays an important role in improving the ecological environment [27,28,29]. Basic molecular research is important to the precise exploitation of *T. succedaneum*, but there is little research in this area. So, the suitable RGs are critical to the molecular biology-related research, while on the contrary, there is no systematic study on the RGs of *T. succedaneum*. Due to this, the selection and identification of reliable RGs will be beneficial to the accuracy of gene quantitative analysis and the related molecular biology research in *T. succedaneum*.

This study evaluated the stable expression of six candidate RGs, according to the *T. succedaneum* transcriptome dataset, and four widely used RGs (*ACT*, *UBQ*, *PP2A2*, and *18S rRNA*). RT-qPCR was used to detect the expression levels of these 10 genes in different tissues (roots, stems, and leaves), abiotic stress (high/low temperature, drought), and hormone stimulation (JA). The ∆Ct value method and three kinds of Excel-based software programs, geNorm [30], Normfinder [31], and BestKeeper [32], were used to evaluate the RT-qPCR results of the 10 candidate RGs, and RefFinder [33] was used to further comprehensively analyze the expression stability of these genes. Overall, this study has excellent benefits and is helpful to the gene function research and molecular breeding of *T. succedaneum*.

## 2. Materials and Methods

### 2.1. Plant Materials and Stress Treatments

The aseptic plantlets of *T. succedaneum* were obtained from the Cell and Tissue Cultures Laboratory of College of Life Sciences, Southwest Forestry University (Kunming 650224, China). They were incubated in an incubator at 24 ℃ with 70% humidity and 90 µmol·m^−2^·s^−1^ light intensity for 12 h of light/12 h of darkness for one week before treatment. For high and low temperature stresses, 4℃ and 35℃ were applied, respectively. For drought treatment, the seedlings were transferred into ½ MS culture medium containing 20% PEG6000 (polyethylene glycol-6000). For hormone stimulation, the seedlings were transferred into a 1/2 MS culture medium containing 25 mM JA [34]. The roots, leaves, and stems of the treated plants were sampled at 6 h, 12 h, and 24 h, respectively, frozen in liquid nitrogen, and stored at −80 ℃ until RNA isolation. Each sample was performed in triplicate biological replicates.

### 2.2. Total RNA Isolation and cDNA Synthesis

Total RNA from leaves, roots, and stems of *T. succedaneum* were isolated by using the TaKaRa MiniBEST Plant RNA Extraction Kit (TAKARA-Bio Inc., Shiga, Japan) according to its manual. The light absorption of RNA at 230 nm (A230), 260 nm (A260), and 280 nm (A280) were determined by using the K5800C spectrophotometer (KAIAO, Beijing, China), and the concentration and purity of RNA were evaluated by the value of A260 and the ratios of 260/280 (1.8–2.1) and 260/230 (2.3–2.6), respectively. RNA samples were assessed by 1.0% agarose gel electrophoresis (AGE). A total of 500 ng RNA from each sample was used for 1st strand cDNA synthesis using Hifair^®^ III Reverse Transcriptase (YEASEN, Shanghai, China) according to the manufacturer’s protocols.

### 2.3. Screening of Candidate RGs and Primers Design

We identified six candidate RGs (Table S1) from the *T. succedaneum* transcriptome data by using the Python library, ERgene (version = 1.2.0) [35]. Four relatively stable traditional RGs were screened according to the gene expression in the transcriptome data of *T. succedaneum*, namely *ACT*, *18S rRNA (18S)*, *UBQ*, and *PP2A2.* The primers of the 10 RGs for RT-qPCR were designed using Primer Preminer 6.0. The LinRegPCR (version = 2021.2) [36] was used to calculate the amplification efficiency and correlation coefficient (R^2^) of each primer pair.

### 2.4. RT-qPCR

RT-qPCR analysis was performed in 96-well plates with the LightCycler^®^ 96 Real-Time PCR Detection System (Roche, Hercules, Switzerland) using Hieff UNICON^®^ Universal Blue qPCR SYBR Green Master Mix (YEASEN, Shanghai, China). The reaction system was prepared in 20 μL volumes containing 1 μL synthesized cDNA, 10 μL Blue qPCR SYBR Green Master Mix, 0.4 μL of each primer (10 μM), and 8.2 μL ddH_2_O. The reactions comprised an initial step at 95 °C for 2 min, followed by 40 denaturation cycles at 95 °C for 10 s and primer annealing at 60 °C for 30 s. Fluorescence intensities were measured for RT-qPCR at the end of each cycle. A melting curve (1 cycle of 95 °C for 10 s, 65 °C for 10 s, and 97 °C for 1 s) was performed directly to check for specific amplification. The experiments were performed in triplicate for each sample. The Ct (cycle threshold) values from RT-qPCR were standardized according to the following Formulas (1) and (2):

(1) $$∆\mathrm{Ct}={\mathrm{Ct}}_{\mathrm{sample}}-{\mathrm{Ct}}_{\min}$$

(2) $$Q=2^{-∆\mathrm{Ct}}$$

∆Ct represented the difference value between Ct_sample_ and Ct_min_; Ct_sample_ indicates the Ct value of each sample in different treatment groups and tissues; Ct_min_ represented the minimum Ct value in each different treatment and tissue; and Q stood for the relative expression of genes.

### 2.5. Assessing the Stability of Candidate Genes Expression

Microsoft Excel 2016 was used to process all the raw Ct values generated by RT-qPCR, and the quartile of Ct values for each RG in all conditions, ∆Ct values and Q values of each RG at the three stages (6 h, 12 h, 24 h) under different treatments and in different tissues were calculated by Excel’s built-in function. All the Q values of each sample at the three stages (6 h, 12 h, and 24 h) under different treatments and in different tissues were imported into GeNorm (https://genorm.cmgg.be/, accessed on 2 May 2022) and NormFinder (https://www.moma.dk/normfinder-software, accessed on 18 May 2022) to overall analysis and identify the stability of RGs. The all the raw Ct values of each sample at the three stages (6 h, 12 h, and 24 h) under different treatments and in different tissues were imported into BestKeeper (https://www.gene-quantification.de/bestkeeper.html, accessed on 22 May 2022) and RefFinder (http://blooge.cn/RefFinder/, accessed on 13 June 2022) to perform an overall analysis and identify the stability of RGs. The data obtained from the experiment were plotted and analyzed by Graphpad Prism 8.0.2 and Microsoft Excel 2016. Adobe Illustrator 2020 was used for chart layout.

## 3. Results

### 3.1. Specificity and Amplification Efficiency of Candidate RGs

We used the ERgene tool to screen six RGs (RGs), *TsRG01*, *TsRG02*, *TsRG03*, *TsRG04*, *TsRG05*, and *TsRG06*, and four traditional RGs (*ACT*, *18S rRNA (18S)*, *UBQ*, and *PP2A2*) that were widely adopted in other plants [7,8,9,10,11,12] (Table 1). Totally, the specific primers of 10 RGs were designed for RT-qPCR, and the specificity of the primers was analyzed based on the melting curve. The results displayed the melting curves of 10 RGs that had a single peak with good repeatability (Figure S1). Furthermore, the RT-qPCR amplification efficiency ranged from 94.12% (*PP2A2*) to 108.57% (*TsRG06*), and the R^2^ of all primers ranged from 0.99897 (*TsRG06*) to 0.99980 (*PP2A2*) (Table 1). These results showed that all primers of RGs met the requirements for RT-qPCR and could be used in further analysis.

### 3.2. Expression Profiling of Candidate RGs

**Figure 1:**
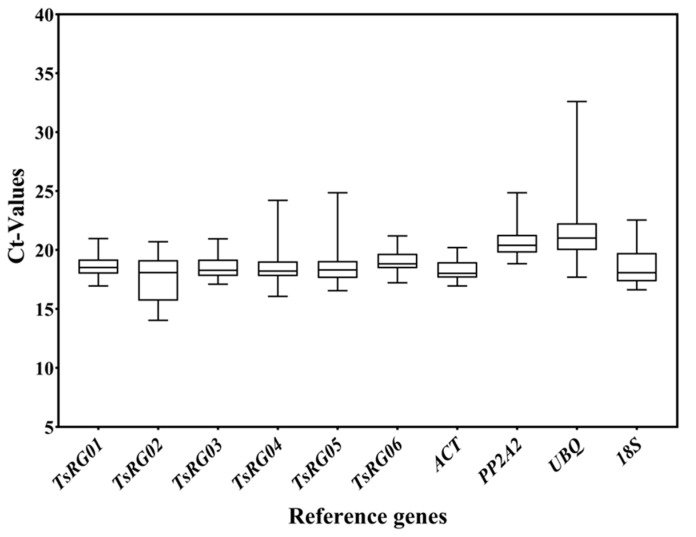
The expression levels of 10 candidate RGs across all experimental samples. The box plot represents the distribution interval of Ct values of 10 RGs in all samples: the upper side represents the upper quartile (75%), the middle line represents the median (50%), the lower side represents the lower quartile (25%), the upper edge represents the maximum value of sample data, and the lower edge represents the minimum value of sample data.

The Ct value represented the expression level of RGs, and the smaller Ct values meant a higher expression level [37]. Moreover, the changes in Ct values reflected the stability of RGs, and the smaller changes in Ct values represent the more stable expression of the genes. The results of expression levels (Ct) of 10 candidate RGs in root, stem, and leaf of *T. succedaneum* under different stresses showed that the Ct values of RGs varied from 14.04 to 32.60, of which the average Ct of *TsRG02* was the smallest (17.62), indicating that its expression level was the highest, and the average Ct of *UBQ* was the highest (21.51), implying that its expression level was the lowest in all samples (Figure 1). According to the Ct values, the expression of the 10 candidate RGs, from high to low, was *TsRG02* > *ACT* > *TsRG04* > *TsRG05* > *TsRG03* > *TsRG01* > *18S* > *TsRG06* > *PP2A2* > *UBQ*. In addition, the range of Ct changes was the smallest in the *ACT* gene (ΔCt = 3.26) and the highest in the *UBQ* gene (ΔCt = 14.91). These results suggested that *TsRG01*, *TsRG03*, *TsRG06*, and *ACT* can be used as RGs in *T. succedaneum* under different stresses and tissues.

### 3.3. The Stability of Candidate RGs Was Analyzed by Genorm Software

The GeNorm program, a Visual Basic application tool for Microsoft Excel, can evaluate gene expression stability (M value), which is the mean pair-wise variation between an individual gene and all other tested control genes [30]. The program ranks the genes based on their M values, and the standard of the selected RGs was also based on the M values. The manual of the GeNorm program recommends M = 1.5 as a threshold [23,38]. So, if the M value of the candidate RGs is more than 1.5, it is not suitable as the reference gene; however, some authors propose the maximum value of 0.5 to obtain more accurate results [23,38]. Meanwhile, the RGs with high M values are less stably expressed, whereas those with low M values are stably expressed [29].

**Figure 2:**
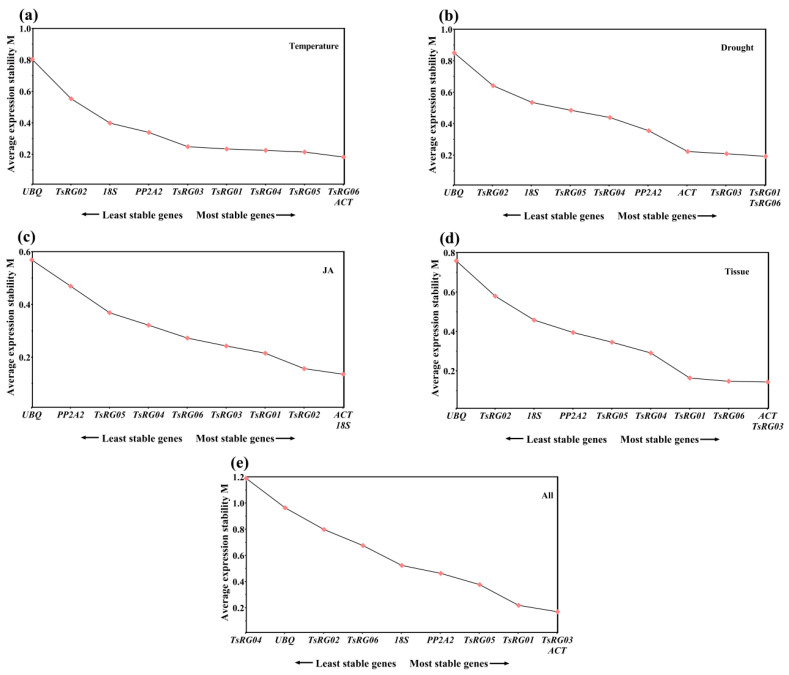
Average expression stability values (M) of 10 candidate RGs under different conditions by geNorm analysis. The direction of the arrows indicates the most and least stable RGs. (**a**) temperature treatments, (**b**) drought treatments, (**c**) JA treatments, (**d**) different tissues, (**e**) all samples.

All the Q values of each RG at the three stages under different treatments and in different tissues were calculated by Genorm to perform an overall analysis and identify the stability of RGs. The results showed that the expression of *TsRG06* and *ACT* (M = 0.183) was more stable than that of other RGs, while *UBQ* (M = 0.803) was the least stable gene (Figure 2a) under the high/low temperature treatment. Under the drought treatment, *TsRG01* and *TsRG06* (M = 0.192) showed the most common and stable expression, while *UBQ* (M = 0.850) was considered the least stable gene (Figure 2b). Under the JA treatment, *18S* and *ACT* (M = 0.136) were more stable and were recommended as RGs to normalize the expression level of target genes, and *UBQ* (M = 0.569) seemed to be inappropriate as a reference gene (Figure 2c). For the samples from different tissues, *ACT* and *TsRG03* (M = 0.144) were regarded as the optimal RGs, while *UBQ* (M = 0.758) was the least stable gene (Figure 2d). Additionally, *TsRG03* and *TsRG06* (M = 0.161) were stable genes, but *TsRG04* (M = 1.079) was the least stable gene in all the samples (Figure 2e).

**Figure 3:**
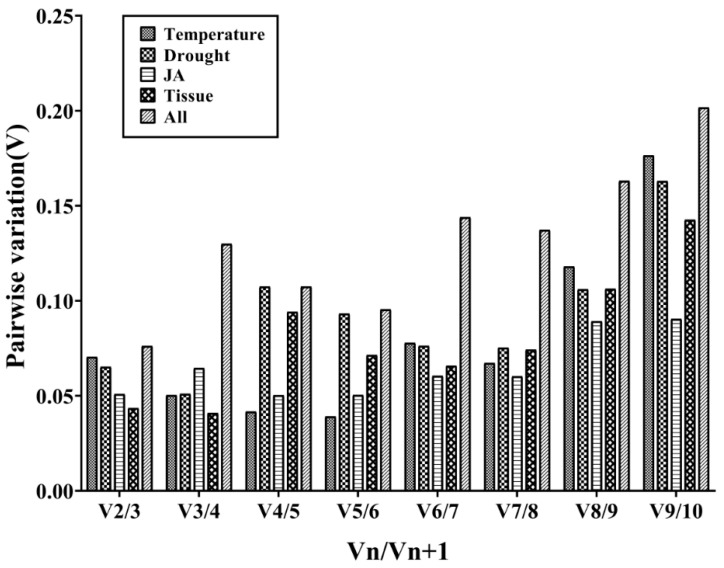
Determination of the optimal number of RGs for normalization by pairwise variation (V) using geNorm. The average pairwise variations (Vn/Vn+1) were analyzed to measure the effect of adding RGs during the RT-qPCR.

Furthermore, the geNorm program can calculate the optimal number of required RGs for obtaining reliable results from RT-qPCR analysis by pairwise variation (V) analysis of RGs. In order to determine the optimal number of candidate RGs required for RT-qPCR data normalization, the geNorm program was used to analyze the pairwise variation (Vn/Vn+1) between the normalization factors (NF) NFn and NFn+1. The geNorm program proposed 0.15 as a cut-off value, which means if Vn/Vn+1 < 0.15, the minimum value of n is the optimal number of genes required [30]. As shown in Figure 3, the V2/3 values in the tissues for temperature stress, drought stress, and JA treatment were less than 0.15, which suggested that the top two RGs were sufficient for accurate normalization. In different tissues, *TsRG03/TsRG06* (V2/V3 = 0.044) was a suitable gene pair for mRNA level normalization. *TsRG06/ACT* (V2/V3 = 0.070) were identified as an appropriate gene set under temperature treatments. Moreover, the gene pair *TsRG01/TsRG06* (V2/V3 = 0.065) was recommended for drought treatment, while *18S/ACT* (V2/V3 = 0.050) were suggested as the RGs under JA treatment. In addition, *TsRG03/TsRG06* (V2/V3 = 0.055) were selected as RGs sets in all the samples (Figure 3).

### 3.4. The Stability of Candidate RGs Was Analyzed by Normfinder Software

NormFinder, another Visual Basic application, was used to evaluate the expression level of 10 candidate RGs; it can automatically calculate the stability value for all candidate RGs on each sample set [31]. The NormFinder software ranks the set of candidate normalization genes according to the expression stability of the candidate RGs. RGs with lower stability values showed fewer varied expressions and a more stable expressed pattern, while genes with higher stability values showed more varied expressions and had the least stable expressed pattern [31]. All the Q values of each RG at the three stages under different treatments and in different tissues were calculated by Normfinder to provide an overall analysis and identify the stability of RGs. According to the results, in the group comprised of samples from temperature stress, drought treatment, and different tissues, *PP2A2* displayed the best stability value, whereas *UBQ* was the most unstable gene. For the JA treatment and all samples, *TsRG03* was the most stable gene, while *TsRG04* was the most unstable gene in all samples (Table 2).

### 3.5. The Stability of Candidate RGs Was Analyzed by Bestkeeper Software

BestKeeper is a program based on Excel that analyzes the stability of the RGs by calculating the standard deviation (SD) and coefficient of variation (CV) of the Ct values of candidate RGs. Therefore, RGs with smaller SD and CV values have more stable expression, and vice versa [32]. All the raw Ct values of each RG at the three stages under different treatments and in different tissues were calculated by Normfinder to provide an overall analysis and identify the stability of RGs. The results indicated that *TsRG01* and *TsRG06* tended to be the most stable genes, as they were listed on the top 2 of the ranks under temperature stress, drought treatments, different tissues, and all samples. On the contrary, *TsRG02* and *UBQ* seemed not to be well-rounded RGs. *TsRG05* and *TsRG04* had values of 0.40 and 0.64 under JA treatments, respectively, and the values showed that *TsRG05* was the most stably expressed RG under JA treatments (Table 3).

### 3.6. The Stability of Candidate RGs Was Analyzed by ∆Ct Value Method

The ΔCt method is based on a comparison of the standard deviation of the ΔCt of the relative expression of paired genes within each sample to identify useful RGs. The reference gene with smaller standard deviations of ΔCt was more stable [39,40]. All the ΔCt values of each RG at the three stages under different treatments and in different tissues were calculated by Excel’s built-in function to perform an overall analysis and identify the stability of RGs. According to ΔCt analysis (Table 4), *TsRG03*, *ACT*, and *TsRG01* were the most stable candidate RGs when all samples were analyzed, as the lowest mean deviation was detected in these genes. Considering the temperature treatment dataset, it appeared that the most stable genes were *TsRG05*, *TsRG04*, and *TsRG03* according to the ΔCt values. For drought samples, the most stable genes were *ACT*, *TsRG03*, and *TsRG06.* For the JA treatment and in different tissues, *TsRG03* was the most stable gene, while *UBQ* was the most unstable gene.

### 3.7. The Stability of Candidate RGs Was Comprehensive Analyzed by RefFinder Software

RefFinder is a web-based online analysis tool that has been used to comprehensively rank the stability of RGs obtained by geNorm, NormFinder, and BestKeeper software and calculate the geometric mean value [33]. The smaller geometric mean stand for the RGs are more stable [33]. However, the results of 10 candidate RGs by three software (geNorm, Normfinde, and BestKeeper) and ∆Ct analysis showed the similarities and differences in stability of RGs in *T. succedaneum*, indicating that different analysis methods achieve different results. Therefore, to obtain a more reasonable ranking order of 10 candidate RGs’ stability, we used the online tool RefFinder to conduct a comprehensive ranking of the results obtained by the above three software programs and ∆Ct analysis. All the raw Ct values of each RG at the three stages under different treatments and in different tissues were calculated by RefFinder to provide an overall analysis and identify the stability of RGs. The results of the RefFinder software showed that *TsRG05* (Table 5A), *TsRG01* (Table 5B and C), and *TsRG03* (Table 5C) were the most stable RGs under high/low temperatures, drought, and JA treatment, respectively. What is more, *TsRG03* was also the most stable reference gene in different tissues (Table 5D). In all samples, *TsRG03* was strongly recommended as a reference gene according to the top geometric mean (ranking values), and *UBQ* was consistently the least reliable gene because of its comprehensive ranking always remained the lowest in all sample sets (Table 5E).

## 4. Discussion

### 4.1. Potential RGs Were Identified from the Transcriptome Data

In this study, six potential RGs were identified based on the *T. succedaneum* transcriptome data using the ERgene tool and the annotation results from nine databases (Gene Ontology (GO), Kyoto Encyclopedia of Genes and Genomes orthology (KOG), Practical Finite-Analytic Method (Pfam), Swissprot, Translation of EMBL (TrEMBL), evolutionary genealogy of genes: Non-supervised Orthologous Groups (eggNOG), nucleotide sequence database (nr), Kyoto Encyclopedia of Genes and Genomes (KEGG). According to the tools and database analyses, *TsRG01*, *TsRG05*, and *TsRG06* belonged to ribosomal RNA and encoded *40S rRNA* (Table S1), and *TsRG03* and *TsRG04* belonged to the elongation factor gene family (*TsRG03* was an *EF-1β*-like gene, and *TsRG04* was an *EF-1α-*like gene). Regrettably, the function of the *TsRG02* gene has not been characterized, and further studies are needed for verification (Table S1). *EF* gene family and ribosomal RNA as the RGs have been reported [7,41,42]. However, thus far, there are no reports on the use of *40S rRNA* as a reference gene. Our results (Table 5) showed that the expression of *40S rRNA* in *T. succedaneum* was stable and could be used as a reference gene. In addition, we found a new reference gene, *TsRG02*, which had a good stability expression when *T. succedaneum* was treated with JA but was unstable under other conditions (Table 5). So, *TsRG02* was specific as an internal reference gene for *T. succedaneum* under high/low temperature and drought stress.

### 4.2. Identification and Selection of RGs

The selection of appropriate RGs is an important preliminary step in the process of gene expression research. Therefore, it is necessary to screen the most stable RGs under different experimental treatments and in different tissues when using RT-qPCR to analyze the target gene expression. A number of studies have shown that the types and stability of RGs vary with different plant species, tissues, stages of development, and biotic/abiotic stress treatments [18]. Therefore, by comparing the analysis results obtained by different RG screening software programs, the most stable RGs can be better determined under diverse situations, including different species, tissues, experimental treatments, etc. Moreover, the experimental error caused by the artificial blind selection of RGs can be eliminated. However, the gene expression stability data obtained by software was not completely consistent in our study, which was caused by different algorithms (Table 5). Therefore, it is essential to use multiple software analyses to increase the reliability of experimental results.

The results of the comprehensive analysis showed that *TsRG05/06*, *TsRG01/06*, and *TsRG01/06* were stable under high/low temperature stress, drought stress, and JA treatment, respectively. *TsRG03* and *ACT* had stable expression in different tissues. In all samples, *TsRG03* and *ACT* were recommended as the suitable RGs for *T. succedaneum*. Our study found that the expression levels of *ACT* and *TsRG03* were stable under JA treatment and in different tissues; therefore, they were recommended as RGs in all samples. However, a previous study has shown that *ACT* was the least stable of the 13 RGs tested in *Cycas elongate* [43], and this is not consistent with our results. So, whether *ACT* could be used as a reference gene in *T. succedaneum* under other treatments needs further experimental verification. On the other hand, *UBQ* belonged to the ubiquitin family gene, which was a kind of traditional reference gene, but its expression level was extremely unstable in all samples in our study. We speculated that this may be due to temperature, drought, and JA stress affecting the dynamic balance between ubiquitination and deubiquitination in *T. succedaneum* cells, just as several studies had shown that *UBQ* was unsuitable for normalization in *Suaeda aralocaspica* and *Camellia sinensis* [44,45]. Furthermore, the expression of *UBQ* was induced by external factors such as high temperature and drought stress in *A. thaliana* [46] and *Vicia faba* [47], respectively. Therefore, it is necessary to screen and evaluate candidate RGs before analyzing the expression of the target gene.

In summary, we screened a series of potential RGs in *T. succedaneum* under different conditions (high/low temperature stress, drought, JA treatment, and different tissues). These results provide alternative RGs for future studies such as exploring the function of genes, correction and normalization of target gene expression, and directed molecular breeding in *T. succedaneum*.

## 5. Conclusions

In this study, three software programs and ΔCt analyses were used to determine the stability of 10 candidate RGs in the different tissues (roots, stems, and leaves) under different stresses in *T. succedaneum*. The results of the comprehensive analysis showed that the stability of the 10 candidate genes under high/low temperature stresses was ranked as *TsRG05 > TsRG06 > ACT > TsRG04 > TsRG01 > PP2A2 > TsRG03 > 18S > TsRG02 > UBQ*, therefore we recommended *TsRG05* and *TsRG06* as the combination of RGs when *T. succedaneum* was under temperature stress. While the stability of 10 genes was ranked as *TsRG01 > TsRG06 > ACT > TsRG03 > PP2A2 > TsRG04 > 18S > TsRG05 > TsRG02 > UBQ* when *T. succedaneum* was treated with PEG600, and *TsRG01* and *TsRG06* were recommended as the better combination of RGs. Furthermore, when *T. succedaneum* was induced by JA, the stability of genes ranked as *TsRG03 > ACT > TsRG02 > TsRG01 > 18S > TsRG06 > TsRG05 > TsRG04 > PP2A2 > UBQ*, so *TsRG03* and *ACT* had good stability as RGs in *T. succedaneum* under JA treatment. Moreover, the stability order of 10 genes in different tissues was *TsRG03 > ACT > PP2A2 > TsRG01 > TsRG04 > TsRG06 > TsRG05 > 18S > TsRG02 > UBQ*, and *TsRG03* and *ACT* were recommended as the better combination of RGs. Lastly, the stability order of 10 genes was ranked as *TsRG03 > ACT > TsRG01 > TsRG05 > TsRG06 > PP2A2 > 18S > TsRG02 > TsRG04 > UBQ* in all conditions, and *TsRG03* and *ACT* were recommended combinations as the RGs. However, according to our results, *UBQ* was not suitable for gene expression analysis in *T. succedaneum*. In any case, our study lays the foundation for further analysis of differential gene expression and molecular mechanisms in *T. succedaneum* in response to different treatments. Moreover, this study is helpful for molecular biology-related research in *T. succedaneum*.

## Figures and Tables

**Table 1:** Primers of candidate RGs.

RGs	Product Size (bp)	Primer Sequences (5′-3′)	R^2^	Efficiency (%)
*TsRG01*	177	F-TTGGGCAGCCGTTGATCTT R-GCTTAGGGTTTCTCCTCTCCTT	0.99943	98.11
*TsRG02*	228	F-GCCTCAAAGCCAGGTAAG R-AGAGTGCCGAAATCATCG	0.99908	102.32
*TsRG03*	134	F-TCTGCTGCCTTCTCATCCTC R-GTGGTACGAATGCGTGTCTT	0.99931	105.47
*TsRG04*	245	F-CTGTCTCACTTGCTGCGGCTAG R-GCACCCAGGCGTACTTGAATGA	0.99913	94.94
*TsRG05*	237	F-CCACAAGTCCAGGGAATGCT R-AGGGAGTGAAGACGGAAACG	0.99898	107.71
*TsRG06*	122	F-TTCACAAAGCGGGTCTCCC R-TTCACTTCCTCACGTGGGTC	0.99897	108.57
*ACT*	188	F-AACTCTCCACCTCGTCCTCC R-TGGACCAGACTCGTCGTACT	0.99958	103.29
*PP2A2*	161	F-CCCTGTGACAATTTGTGGCG R-TAAGGGCCACTAACAGCGTG	0.99980	94.12
*UBQ*	200	F-GGGTCCTCCCATCCTCAAGT R-AACTCTCCACCTCGTCCTCC	0.99901	94.13
*18S*	209	F-GAAGAGCATCCAGTGCTT R-TGCATCGGTGAGATCACG	0.99927	101.17

**Table 2:** Stability of candidate RGs calculated by NormFinder under different conditions.

Rank	Temperature		Drought		JA		Tissues		All	
	Gene	Stability	Gene	Stability	Gene	Stability	Gene	Stability	Gene	Stability
1	*PP2A2*	0.12	*PP2A2*	0.27	*TsRG03*	0.05	*PP2A2*	0.11	*TsRG03*	0.08
2	*18S*	0.22	*18S*	0.38	*TsRG02*	0.11	*TsRG04*	0.29	*ACT*	0.13
3	*TsRG05*	0.24	*ACT*	0.39	*ACT*	0.2	*TsRG05*	0.34	*TsRG01*	0.13
4	*TsRG04*	0.26	*TsRG03*	0.43	*TsRG06*	0.20	*18S*	0.35	*TsRG05*	0.30
5	*TsRG03*	0.34	*TsRG04*	0.45	*TsRG01*	0.21	*TsRG03*	0.39	*PP2A2*	0.36
6	*ACT*	0.42	*TsRG05*	0.47	*18S*	0.28	*ACT*	0.44	*18S*	0.48
7	*TsRG06*	0.45	*TsRG06*	0.47	*TsRG04*	0.38	*TsRG01*	0.46	*TsRG06*	1.01
8	*TsRG01*	0.46	*TsRG01*	0.49	*TsRG05*	0.57	*TsRG06*	0.46	*TsRG02*	1.08
9	*TsRG02*	0.99	*TsRG02*	0.86	*PP2A2*	0.76	*TsRG02*	0.88	*UBQ*	1.59
10	*UBQ*	1.76	*UBQ*	1.62	*UBQ*	0.90	*UBQ*	1.42	*TsRG04*	2.00

**Table 3:** Expression stability values of candidate RGs under different conditions were calculated by BestKeeper.

Samples		1	2	3	4	5	6	7	8	9	10
Temperature	gene	*TsRG06*	*TsRG01*	*ACT*	*TsRG03*	*TsRG04*	*TsRG05*	*PP2A2*	*18S*	*TsRG02*	*UBQ*
	SD	0.72	0.74	0.83	0.86	0.93	0.94	1.27	1.40	1.65	2.05
	CV	3.72	3.92	4.52	4.57	5.01	5.03	6.09	7.42	9.15	9.35
Drought	gene	*TsRG01*	*TsRG06*	*TsRG03*	*TsRG04*	*ACT*	*TsRG05*	*PP2A2*	*18S*	*TsRG02*	*UBQ*
	SD	0.65	0.65	0.66	0.74	0.74	0.74	0.95	1.22	1.47	1.89
	CV	3.47	3.42	3.56	3.99	4.05	4.00	4.55	6.40	8.17	8.45
JA	gene	*TsRG05*	*TsRG04*	*TsRG06*	*TsRG01*	*TsRG03*	*ACT*	*TsRG02*	*18S*	*PP2A2*	*UBQ*
	SD	0.40	0.64	0.67	0.74	0.77	0.92	0.93	0.97	1.30	1.43
	CV	2.33	3.80	3.71	4.17	4.27	5.13	6.09	5.50	6.11	7.28
Tissues	gene	*TsRG01*	*TsRG06*	*TsRG03*	*ACT*	*TsRG05*	*TsRG04*	*PP2A2*	*18S*	*TsRG02*	*UBQ*
	SD	0.67	0.70	0.72	0.73	0.81	0.84	1.05	1.32	1.51	1.82
	CV	3.59	3.64	3.88	3.99	4.33	4.50	5.05	6.99	8.41	8.36
All	gene	*TsRG01*	*TsRG06*	*TsRG03*	*ACT*	*TsRG05*	*TsRG04*	*PP2A2*	*18S*	*TsRG02*	*UBQ*
	SD	0.72	0.73	0.75	0.76	0.85	0.88	1.09	0.33	1.71	1.79
	CV	3.88	3.83	4.04	4.19	4.60	4.80	5.23	7.12	9.70	8.31

**Table 4:** Expression stability of 10 candidate RGs under different conditions were calculated by ∆Ct.

Rank	Temperature		Drought		JA		Tissues		All	
	Gene	Stability	Gene	Stability	Gene	Stability	Gene	Stability	Gene	Stability
1	*TsRG05*	0.57	*ACT*	0.66	*TsRG03*	0.42	*TsRG03*	0.59	*TsRG03*	0.85
2	*TsRG04*	0.59	*TsRG03*	0.67	*TsRG02*	0.44	*PP2A2*	0.62	*ACT*	0.87
3	*TsRG03*	0.61	*TsRG06*	0.68	*TsRG01*	0.44	*TsRG04*	0.62	*TsRG01*	0.87
4	*TsRG06*	0.62	*PP2A2*	0.70	*ACT*	0.44	*ACT*	0.62	*TsRG05*	0.93
5	*ACT*	0.63	*TsRG01*	0.70	*TsRG06*	0.46	*TsRG06*	0.62	*PP2A2*	0.93
6	*TsRG01*	0.65	*TsRG04*	0.76	*18S*	0.48	*TsRG01*	0.62	*18S*	0.97
7	*PP2A2*	0.68	*18S*	0.77	*TsRG04*	0.54	*TsRG05*	0.65	*TsRG06*	1.32
8	*18S*	0.71	*TsRG05*	0.79	*TsRG05*	0.66	*18S*	0.71	*TsRG02*	1.34
9	*TsRG02*	1.17	*TsRG02*	1.07	*PP2A2*	0.84	*TsRG02*	1.05	*UBQ*	1.72
10	*UBQ*	1.80	*UBQ*	1.68	*UBQ*	0.97	*UBQ*	1.47	*TsRG04*	2.09

**Table 5:** The stability ranking order of 10 candidate RGs were calculated by RefFinder.

A. Ranking Order under High/Low Temperature Stress (Better–Good–Average)						
Ranking	∆Ct	geNorm	Normfinder	BestKeeper	Comprehensive ranking	Ranking values
1	*TsRG05*	*TsRG06|ACT*	*PP2A2*	*TsRG01*	*TsRG05*	2.59
2	*TsRG04*	*-*	*18S*	*TsRG06*	*TsRG06*	2.74
3	*TsRG03*	*TsRG05*	*TsRG05*	*ACT*	*ACT*	3.08
4	*TsRG06*	*TsRG04*	*TsRG04*	*TsRG04*	*TsRG04*	3.36
5	*ACT*	*TsRG01*	*TsRG03*	*TsRG05*	*TsRG01*	3.94
6	*TsRG01*	*TsRG03*	*ACT*	*TsRG03*	*PP2A2*	4.30
7	*PP2A2*	*PP2A2*	*TsRG06*	*PP2A2*	*TsRG03*	4.82
8	*18S*	*18S*	*TsRG01*	*18S*	*18S*	5.66
9	*TsRG02*	*TsRG02*	*TsRG02*	*TsRG02*	*TsRG02*	9.00
10	*UBQ*	*UBQ*	*UBQ*	*UBQ*	*UBQ*	10.00
B. Ranking Order under drought stress (Better–Good–Average)						
Ranking	∆Ct	geNorm	Normfinder	BestKeeper	Comprehensive ranking	Ranking values
1	*ACT*	*TsRG06|TsRG01*	*PP2A2*	*TsRG01*	*TsRG01*	2.51
2	*TsRG03*	*-*	*18S*	*TsRG06*	*TsRG06*	2.55
3	*TsRG06*	*TsRG03*	*ACT*	*TsRG03*	*ACT*	2.78
4	*PP2A2*	*ACT*	*TsRG03*	*TsRG04*	*TsRG03*	2.91
5	*TsRG01*	*PP2A2*	*TsRG04*	*ACT*	*PP2A2*	3.44
6	*TsRG04*	*TsRG04*	*TsRG05*	*TsRG05*	*TsRG04*	5.18
7	*18S*	*TsRG05*	*TsRG06*	*PP2A2*	*18S*	5.47
8	*TsRG05*	*18S*	*TsRG01*	*18S*	*TsRG05*	6.70
9	*TsRG02*	*TsRG02*	*TsRG02*	*TsRG02*	*TsRG02*	9.00
10	*UBQ*	*UBQ*	*UBQ*	*UBQ*	*UBQ*	10.00
C. Ranking Order under JA stress (Better–Good–Average)						
Ranking	∆Ct	geNorm	Normfinder	BestKeeper	Comprehensive ranking	Ranking values
1	*TsRG03*	*ACT|18S*	*TsRG03*	*TsRG05*	*TsRG03*	2.24
2	*TsRG02*	*-*	*TsRG02*	*TsRG04*	*ACT*	2.91
3	*TsRG01*	*TsRG02*	*ACT*	*TsRG06*	*TsRG02*	3.03
4	*ACT*	*TsRG01*	*TsRG06*	*TsRG01*	*TsRG01*	3.94
5	*TsRG06*	*TsRG03*	*TsRG01*	*TsRG03*	*18S*	4.12
6	*18S*	*TsRG06*	*18S*	*ACT*	*TsRG06*	4.36
7	*TsRG04*	*TsRG04*	*TsRG04*	*TsRG02*	*TsRG05*	4.76
8	*TsRG05*	*TsRG05*	*TsRG05*	*18S*	*TsRG04*	5.12
9	*PP2A2*	*PP2A2*	*PP2A2*	*PP2A2*	*PP2A2*	9.00
10	*UBQ*	*UBQ*	*UBQ*	*UBQ*	*UBQ*	10.00
D. Ranking Order under different tissues (Better–Good–Average)						
Ranking	∆Ct	geNorm	Normfinder	BestKeeper	Comprehensive ranking	Ranking values
1	*TsRG03*	*TsRG03|ACT*	*PP2A2*	*TsRG01*	*TsRG03*	1.97
2	*PP2A2*	*-*	*TsRG04*	*TsRG06*	*ACT*	3.13
3	*TsRG04*	*TsRG06*	*TsRG05*	*TsRG03*	*PP2A2*	3.15
4	*ACT*	*TsRG01*	*18S*	*ACT*	*TsRG01*	3.6
5	*TsRG06*	*TsRG04*	*TsRG03*	*TsRG05*	*TsRG04*	3.66
6	*TsRG01*	*TsRG05*	*ACT*	*TsRG04*	*TsRG06*	3.94
7	*TsRG05*	*PP2A2*	*TsRG01*	*PP2A2*	*TsRG05*	5.01
8	*18S*	*18S*	*TsRG06*	*18S*	*18S*	6.73
9	*TsRG02*	*TsRG02*	*TsRG02*	*TsRG02*	*TsRG02*	9.00
10	*UBQ*	*UBQ*	*UBQ*	*UBQ*	*UBQ*	10.00
E. Ranking Order under all samples (Better–Good–Average)						
Ranking	∆Ct	geNorm	Normfinder	BestKeeper	Comprehensive ranking	Ranking values
1	*TsRG03*	*TsRG03|ACT*	*TsRG03*	*TsRG01*	*TsRG03*	1.32
2	*ACT*	*-*	*ACT*	*TsRG06*	*ACT*	2.00
3	*TsRG01*	*TsRG01*	*TsRG01*	*TsRG03*	*TsRG01*	2.28
4	*TsRG05*	*TsRG05*	*TsRG05*	*ACT*	*TsRG05*	4.23
5	*PP2A2*	*PP2A2*	*PP2A2*	*TsRG05*	*TsRG06*	5.12
6	*18S*	*18S*	*18S*	*TsRG04*	*PP2A2*	5.44
7	*TsRG06*	*TsRG06*	*TsRG06*	*PP2A2*	*18S*	6.45
8	*TsRG02*	*TsRG02*	*TsRG02*	*18S*	*TsRG02*	8.24
9	*UBQ*	*UBQ*	*UBQ*	*TsRG02*	*TsRG04*	8.80
10	*TsRG04*	*TsRG04*	*TsRG04*	*UBQ*	*UBQ*	9.24

## Data Availability

Data supporting this study can be obtained by contacting the corresponding author upon reasonable request.

## Supplementary Materials

The following supporting information can be downloaded at: https://www.mdpi.com/article/10.3390/genes13122396/s1, Figure S1: The melting curve analysis of real-time quantitative PCR (RT-qPCR) amplification of 10 candidate RGs in *T. succedaneum*; Table S1: Database annotation of six RGs from transcriptome data.
