# Supplementary material for: Identification of Reliable Reference Genes under Different Stresses and in Different Tissues of Toxicodendron succedaneum
Source: Genes (Basel). 2022 Dec 17;13(12):2396. doi: 10.3390/genes13122396 (PMC9778191; doi:10.3390/genes13122396)
Supplement: Supplementary file 1 [file genes-13-02396-s001.zip › Figure S1.pdf]

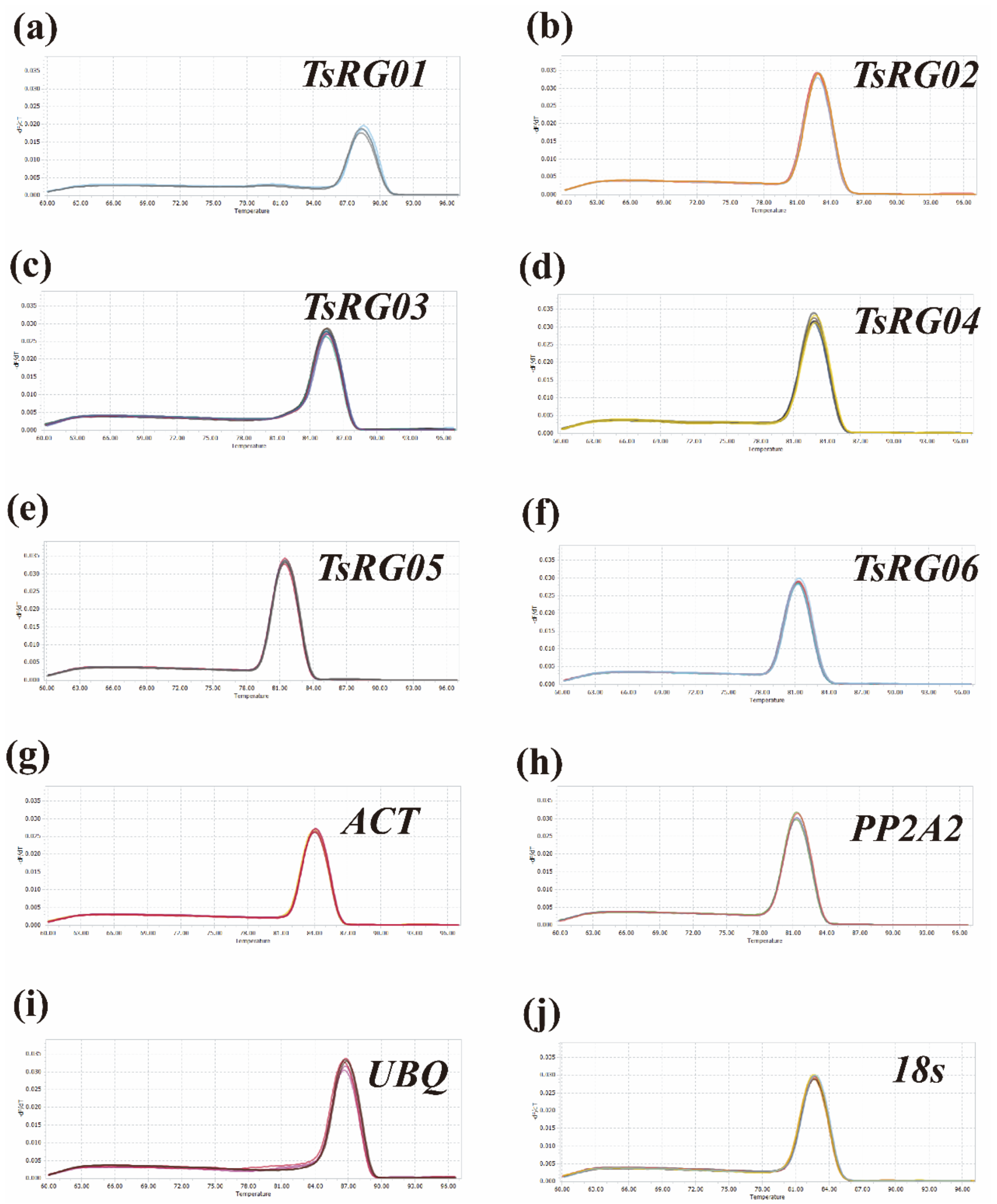

Figure S1: The melting curve analysis of real-time quantitative PCR (RT-qPCR) amplification of 10 candidate RGs in *T. succedaneum*. The X-axis is the temperature (℃), and the Y-axis is the differential value of the signal (-dF/dT). Different colored curves represent different samples (a): the melting curve of *TsRG01*; (b): the melting curve of *TsRG02*; (c): the melting curve of *TsRG03*; (d): the melting curve of *TsRG04*; (e): the melting curve of *TsRG05*; (f): the melting curve of *TsRG06*; (g): the melting curve of *ACT*; (h): the melting curve of *PP2A2*; (i): the melting curve of *UBQ*; (j): the melting curve of *18S rRNA*.
